# Unraveling nonadiabatic ionization and Coulomb potential effect in strong-field photoelectron holography

**DOI:** 10.1038/srep28392

**Published:** 2016-06-22

**Authors:** Xiaohong Song, Cheng Lin, Zhihao Sheng, Peng Liu, Zhangjin Chen, Weifeng Yang, Shilin Hu, C. D. Lin, Jing Chen

**Affiliations:** 1 Department of Physics, College of Science, Shantou University, Shantou, Guangdong 515063, People’s Republic of China; 2 HEDPS, Center for Applied Physics and Technology, Peking University, Beijing 100084, People’s Republic of China; 3 Institute of Applied Physics and Computational Mathematics, P. O. Box 8009, Beijing 100088, People’s Republic of China; 4 J.R.Macdonald Laboratory, Physics Department, Kansas State University, Manhattan,Kansas 66506-2604, USA

## Abstract

Strong field photoelectron holography has been proposed as a means for interrogating the spatial and temporal information of electrons and ions in a dynamic system. After ionization, part of the electron wave packet may directly go to the detector (the reference wave), while another part may be driven back and scatters off the ion(the signal wave). The interference hologram of the two waves may be used to extract target information embedded in the *collision process.* Unlike conventional optical holography, however, propagation of the electron wave packet is affected by the Coulomb potential as well as by the laser field. In addition, electrons are emitted over the whole laser pulse duration, thus multiple interferences may occur. In this work, we used a generalized quantum-trajectory Monte Carlo method to investigate the effect of Coulomb potential and the nonadiabatic subcycle ionization on the photoelectron hologram. We showed that photoelectron hologram can be well described only when the effect of nonadiabatic ionization is accounted for, and Coulomb potential can be neglected only in the tunnel ionization regime. Our results help paving the way for establishing photoelectron holography for probing spatial and dynamic properties of atoms and molecules.

Atomic photoionization under intense laser irradiation is a fundamental process in strong field light-matter interaction. Since above-threshold ionization (ATI) was firstly observed more than thirty years ago^1^, through experimental observations and theoretical efforts our understanding of the underlying physics of laser-atom interactions^2^,^3^ has greatly advanced. In recent years, with the availability of new long wavelength laser and high resolution electron spectrometer, photoelectron spectra from some recent experiments have revealed a number of surprises. Besides the familiar ATI peaks, new additional “peaks or fringes” have been observed in the two-dimensional electron momentum spectra. These new features, usually are called by some new acronyms or simply by “structures”, appear to be quite general, as they are nearly independent of the target atoms or molecules, but they are dependent on the laser wavelength, intensity and sometimes also on the pulse duration. Among these photoelectrons are the so-called “low-energy structures” (LES) at a few or sub-eV’s or the “very low-energy structure” (VLES) at a few meV’s above the ionization threshold^4^,^5^,^6^,^7^,^8^,^9^,^10^,^11^. In most cases the widely used strong field approximation (SFA) is incapable of interpreting these observations. For such low-energy electrons, it is intuitively clear that a quantitative theory would require the incorporation of Coulomb potential from the ion core. On the other hand, there are higher energy features^12^,^13^,^14^,^15^,^16^,^17^,^18^,^19^ that lie close to the so-called 2*U*_*p*_ cutoff (*U*_*p*_ is the ponderomotive energy or the averaged quiver energy of a free electron in the laser field, 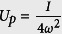 where *I* the laser intensity and *ω* the angular frequency). Among them we will focus on the so-called “side lobes” observed in the photoelectron momentum distribution (PMD). Such side lobes were observed in the PMD of metastable xenon atoms ionized with intense 7000 nm free-electron lasers^12^. They have been further observed at other wavelengths in other experiments or found in numerical calculations. These side lobes were interpreted as analogous to optical holograms, resulting from the interference of electron wave packet from direct ionization (reference wave) with laser-driven rescattered electron wave packet (the signal wave) after ionization. Like holography, such interference may encode target structure information that is embedded from the collision process.

Atomic photoionization in intense laser fields are often categorized into two regimes according to whether the Keldysh parameter *γ*^20^ is less (tunnel ionization) or greater (multiphoton ionization) than 1.0. Here 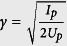, with *I*_*p*_ being the ionization potential. In the tunneling limit, where *γ* approaches zero, the electron spectrum is considered as resulting from the superposition of complex electron wave packets generated at each point in time of the laser pulse. In this quasistatic picture, the electron reaching the detector will reflect vast number of different types of interference and may not be easily tractable. However, in certain circumstances, simple interference patterns like the “side lobes” are observable in the photoelectron momentum distributions. Indeed, the “side lobes” can be qualitatively explained in terms of the interference of electrons following two different quantum paths^12^,^19^ where the trajectory of each path is calculated classically and the phase from the semiclassical action. In fact, one of the two paths is from the direct wave where the electron leaves the laser field directly, the other path is a signal wave where the electron interacts (or collides) with the atomic ion once (the signal wave) before it reaches the detector. While numerical solution of the time-dependent Schrödinger equation (TDSE) is able to quantitatively reproduce the experimental observation^12^,^13^,^14^,^16^,^18^,^19^ mostly, the method is incapable of separating out the direct wave from the signal wave, nor is any other quantum theory as far as we know. While a quantal Coulomb-corrected SFA theory (CCSFA)^12^ has some success in reproducing the “side lobe” patterns, quantitatively the CCSFA results do not compare well with experimental data nor with TDSE results.

To reproduce the observed side lobes quantitatively, here we used a generalized quantum-trajectory Monte Carlo (GQTMC) method to simulate photoelectron spectra. GQTMC is an extension of the previously used quantum-trajectory Monte Carlo (QTMC) method, which in turn was extended from the classical-trajectory Monte Carlo method^21^,^22^,^23^ by including quantum interference effect after tunneling^24^. The QTMC method has been widely used to interpret photoelectron spectra in recent years with great success. However, the QTMC method treats ionization under the quasistatic approximation and may agree with experiments in the deep tunneling regimes only, i.e., for 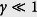
20.

In strong field physics it has always been of great interest to look for signature of nonadiabatic effect, or the so-called nonadiabatic tunneling^25^,^26^ in ionization^27^,^28^,^29^,^30^,^31^, in view that most experiments have been carried out in the transition regime where *γ* ~ 1. Because side lobes have been observed both in the tunneling^12^ and the nonadiabatic tunneling regimes^15^,^16^, in this work we used GQTMC and QTMC to study the side lobes. We have found that GQTMC simulations are always in much better agreement with experiment and with TDSE results. On the other hand, Coulomb effect is significant only in the multiphoton ionization regime.

## Results

### Comparison of simulations with experiment

**Figure 1:**
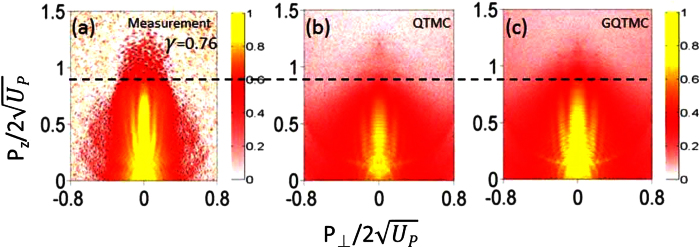
Comparison of experimental two-dimensional photoelectron momentum distributions with calculations, for the metastable 6s state (*I*_*p*_ = 0.14 a.u.) of xeon atom by lasers of wavelength of 7000 nm. (**a**) Experimental data from ref. 12 at *I* = 7.1 × 10^11 ^W/cm^2^; (**b**) QTMC simulation, the laser pulse envelope is half-trapezoidal, constant for the first four cycles and ramped off linearly within the last two cycles, and the peak intensity is *I* = 9.1 × 10^11 ^W/cm^2^; (**c**) same as (**b**) but for GQTMC. The horizontal dashed line is the cutoff energy of the side lobe. The simulations included laser intensity distributions in the focused volume.

Figure 1 shows the experimental PMD obtained from Huismans *et al*.^12^ where PMD was reported for ionization of the metastable 6 s electron of Xe by a 7000 nm laser with peak intensity of *I* = 7.1 × 10^11 ^W/cm^2^, corresponding to *γ* = 0.76. In the QTMC and GQTMC calculations, like in experiment, we have integrated electron signals from the whole focal volume. To our knowledge, this is the first time that the data of Huismans *et al*. are compared to simulations that include volume integration. We found that the experimental data best agree with GQTMC if the peak laser intensity is 9.1 × 10^11 ^W/cm^2^, about 30 percent higher than the value cited in the experiment. The effect of focal averaging lies in that, after focal averaging: (i) the ring interference fringes at large final longitudinal momentum become less visible; (ii) the higher side lobes (i.e., the 2nd and 3rd holographic interferences) would become clearer. Note that the cutoff energy in the GQTMC simulation is in good agreement with the experimental data, while simulation with the same laser intensity using QTMC always has a lower cutoff energy. The side lobes are clearly seen in all three frames. Besides the main lobe along the polarization axis, there are two lobes on each side of the axis, with the outer 2nd lobe being weaker. The simulations also show one strong arc on each side nearly perpendicular to the polarization axis, which can be seen in the experimental data (the red arc in Fig. 1(a)). This is the transverse fork/off-axis low-energy structure found by M. Möller *et al*.^32^.

### Nonadiabatic effects

**Figure 2:**
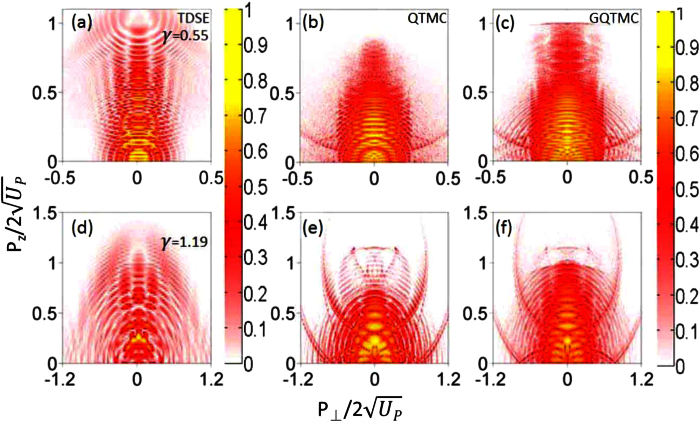
Comparison of two-dimensional photoelectron momentum spectra of Xenon atom from the ground state using TDSE, QTMC and GQTMC. Upper row: *γ* = 0.55, *I* = 7.0 × 10^13 ^W/cm^2^. The momentum in the vertical scale is normalized with respect to the cutoff momentum 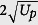. Lower row: *γ* = 1.19, *I* = 1.5 × 10^13 ^W/cm^2^. In TDSE simulation, we use sine-square pulse with duration of 12 optical cycles. In QTMC and GQTMC simulations, for convenience of analysis, the laser pulse envelope is half-trapezoidal, constant for the first six cycles and ramped off linearly in the last four cycles. Laser wavelength: *λ* = 1700 nm. The results show that side lobes from QTMC are less well developed than those obtained from TDSE and GQTMC. The cutoff energy of the side lobes from GTMC is lower than from the other two, illustrating that nonadiabatic ionization effect is not negligible even for *γ* = 0.55.

Figure 2 shows that nonadiabatic ionization is important for describing side lobes correctly. Two laser intensities are used, one for *γ* = 0.55 and another for *γ* = 1.19. Xeon atom in the ground state was used and QTMC and GQTMC were used together with solutions from TDSE^17^,^18^. One finds that side lobes are seen more clearly in TDSE and GQTMC than in QTMC. The cutoff energy in QTMC also tends to be lower than the other two. These single intensity results demonstrate that accurate description of side lobes needs to account for nonadiabatic ionization effect. This is qualitatively understood by the fact that side lobes occur for higher energy direct electrons. In the static theory, ionization rate is very small when the field is weak where the vector potential is large. The nonadiabatic effect enhances ionization yield especially when the laser field is near zero. The functionals in the exponential dependence in the Yudin-Ivanov formula would draw contributions from higher field component. Thus effect of nonadiabatic tunneling is to enhance formation of side lobes. In Fig. 2, these observations hold for both tunneling and multiphoton regimes.

**Figure 3:**
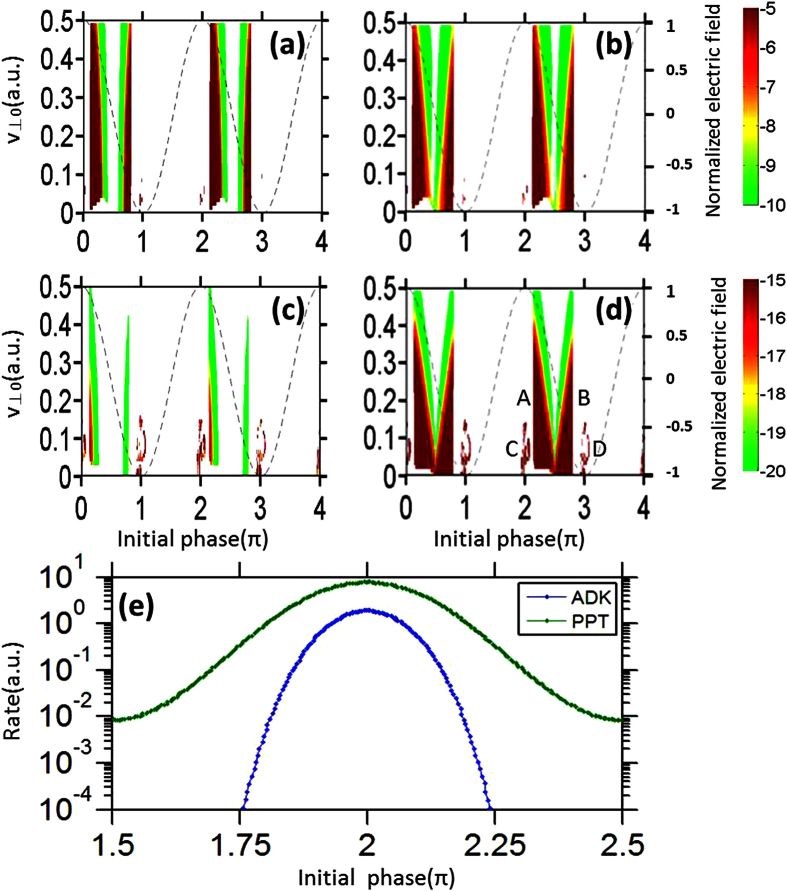
Distributions of the initial transverse velocity and the initial ionization phase of the laser. Left column: QTMC simulations; right column: GQTMC simulations. The dash line is the laser electric field. Upper row: *γ* = 0.55. Lower row: *γ* = 1.19. The simulation parameters are the same as in Fig. 2(e) comparison of total ionization rate using ADK theory and the PPT model for *γ* = 1.19, vs the phase of the laser field. Difference due to nonadiabatic ionization is much larger when the laser electric field is small, e.g., at 1.75 or 2.25 *π* of Fig. 3(e).

To further explore nonadiabatic ionization effect we performed quantum trajectories analysis of the PMD for both *γ* = 0.55 and *γ* = 1.19, as shown in Fig. 3. We analyze the normalized momentum interval of 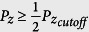, with 
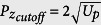
 being the momentum corresponding to the 2*U*_*p*_ cutoff energy. In this region, the momentum distributions are dominated by the side lobe structure. The QTMC and GQTMC models offer the opportunity to trace back the initial transverse velocity and the initial laser phase for each electron contributing to a given momentum distribution spot. By comparing these two models, the influence of nonadiabatic ionization can be unraveled. In Fig. 3(d), over half of an optical period (laser phase from 2*π* to 3*π*), we used A, B, C, D to denote the ionization phase vs the distribution of the initial transverse velocity. The electrons generated at each of such a half optical cycle will interfere. Similar plots are also shown in (a) to (c). The differences among these frames are due to the degree of nonadiabaticity in tunnel ionization.

For *γ* = 0.55, the initial phase distributions for QTMC and GQTMC models are very similar, but the areas A and B calculated by GQTMC are slightly wider than those by QTMC. For the case of *γ* = 1.19, dramatic change occurs in these two areas. The much broader width in GQTMC as compared to QTMC reflects the subcycle nonadiabatic ionization dynamics where ionization does not follow the instantaneous electric field of the laser pulse, but “spreads” out over a broader time interval, thus resulting in a much sharper contrast in subcycle ionization dynamics in the multiphoton ionization regime, as clearly seen between (c) and (d) where *γ* = 1.19. Figure 3(e) compares the subcycle ionization rates over one quarter cycle according to ADK^25^,^33^ model vs the PPT model^34^. Note in Fig. 3(e), the ionization includes all energies of the photoelectrons, while Fig. (3a–d) include only higher electron energies described in the previous paragraph.

**Figure 4:**
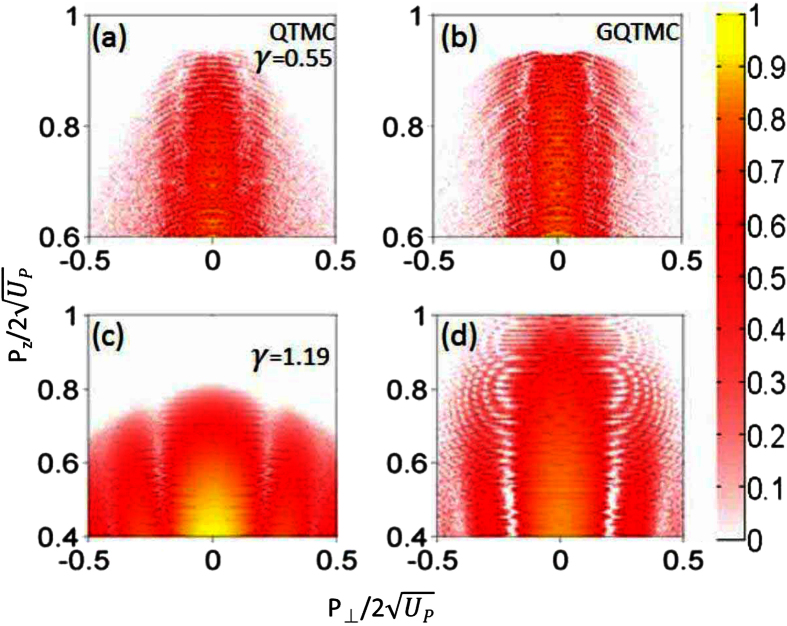
Comparison of the final PMD due to ionization from area A (see Fig. 3d) calculated by the QTMC and GQTMC for *γ* = 0.55 and 1.19, respectively. The parameters are the same as in Fig. 2.

It has been demonstrated that side lobes originate from interference between direct and rescattered electron wave packets that were initiated within the same quarter cycle of the laser field^12^, i.e., from area A in Fig. 3(d). To demonstrate this, we show in Fig. 4 the calculated PMD for electrons emitted from area A in each of the four cases in Fig. 3. Consistent with the previous SFA and classical calculations^12^,^19^, electrons emitted from area A indeed yield side lobes as holographic interference patterns (see Fig. 4). For *γ* = 0.55, both QTMC and GQTMC methods reproduce well the side lobes seen from the TDSE calculation (see Fig. 2). For *γ* = 1.19, the QTMC method underestimates the cutoff energy of the side lobes. Further examination of the initial velocity distributions of electrons emitted from area A for *γ* = 1.19 (see Fig. 3(c,d)), one finds that the mismatch between QTMC and GQTMC is much more severe when the instantaneous electric field is close to zero. The quasi-static ADK theory severely underestimates the ionization yield in this part of the laser field when *γ* ~ 1. Due to nonadiabatic tunneling, substantial ionization can occur even when the instantaneous electric field is weak^25^,^26^. Since subcycle ionization dynamics is well described by Eq. (4)^25^ which is the basis of our GQTMC model, the PMD’s calculated via GQTMC are able to describe accurately the side lobes observed in the experiments or from the TDSE calculations over a large range of *γ*.

Next consider ionization from other laser phase areas B, C, and D. For example, ionization from C and D occurs near the field crest, they show no evidence of nonadiabatic tunneling ionization, and QTMC and GQTMC results are essentially identical, see Fig. 3. Ionization from C and D becomes significantly more important for *γ* = 1.19 in the multiphoton ionization regime. Furthermore, it has been demonstrated that electron trajectories in area C correspond to a form of transverse backward-scattering driven by the Coulomb field, which induces a totally different interference structure from that of the side lobe electrons^35^. In the QTMC simulation, since the cutoff energy induced by area A is low, interference fringes induced by electrons from area C can be seen beyond the side lobes, see Fig. 2(e). However for GQTMC simulation, these interference fringes are only observable at large transverse final momentum where the side lobes are much weaker (see Fig. 2(f)). It should be mentioned here that, similar to area B, electrons from area D, which come from the negative electric field, only contribute to the background of the fringes.

### Long-range Coulomb potential effects

**Figure 5:**
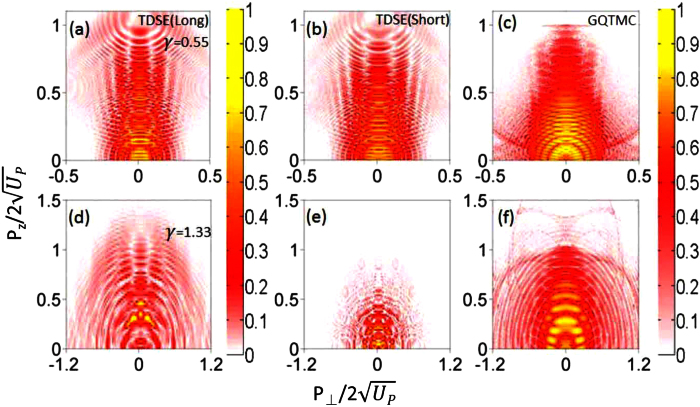
Effect of long range Coulomb potential on the side lobes. Upper row for *γ* = 0.55, *I* = 5.0 × 10^13 ^W/cm^2^, *λ* = 2000 nm. Lower row for *γ* = 1.33, *I* = 1.2 × 10^13 ^W/cm^2^, *λ* = 1700 nm. Left column: with long-range Coulomb potential. Middle and right column: with short-range potential. Side lobes seen with or without Coulomb potential for *γ* = 0.55. Weak side lobes can be seen in TDSE with Coulomb potential, but not for short range potential for both TDSE and GQTMC, for *γ* = 1.33. In the multiphoton ionization regime, Coulomb potential is needed to incur large angle collision for the direct process for the side lobe to appear.

In the following, we explore the role of Coulomb potential in the holographic interference structures. We calculated PMDs by solving TDSE and by GQTMC for model atoms with a long-range Coulomb potential, and with a short-range potential^36^, for various laser intensities and wavelengths shown in Fig. 5. For *γ* = 0.55, the side lobes are clearly visible for solutions from TDSE, with Coulomb potential (a) or with short-range potential (b). Using GQTMC, though the side lobes are weaker for short range potential (c) than for long range potential (see Fig. 2(f)), they are still visible. In contrast, for *γ* = 1.33, the interference pattern can be seen from TDSE with Coulomb potential (see Fig. 5(d)), but for short-range potential, side lobes are hardly discernable in both TDSE (see Fig. 5(e)) and GQTMC simulations (see Fig. 5(f)). We thus demonstrated that Coulomb potential plays a significant role for observing side lobes in the nonadiabatic regime, but not in the adiabatic tunneling regime.

**Figure 6:**
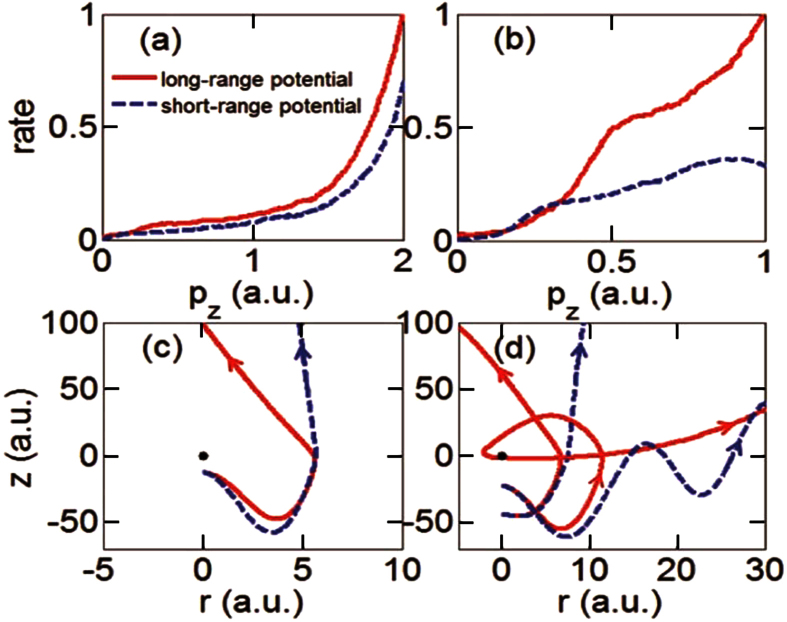
Effect of Coulomb potential vs short-range potential on the final longitudinal momentum distribution of the rescattering electrons for *γ* = 0.55 (left column) and 1.33 (right column), respectively. Typical electron trajectories are shown for these two regimes also. Note that z is the longitudinal direction and r is the transverse direction. The parameters are the same as in Fig. 5.

To explain these observations, we analyze the statistics of the longitudinal momentum of the rescattering electrons, for both long-range and short-range potentials, see Fig. 6. For *γ* = 0.55, the long-range potential tends to scatter electrons with larger longitudinal momentum (Fig. 6(a)), but the difference with a short-range potential case is not large. Moreover, the probability of scattering with large change of transverse momentum, which is essential for the formation of interference fringe, is also reduced in the short-range potential (see typical trajectories shown in Fig. 6(c)). Therefore, in GQTMC simulation for *γ* = 0.55, the fringes become slightly less apparent in the short-range potential (Fig. 5(c)) than that in the long-range potential (Fig. 2(c)) . More interestingly, in the nonadiabatic tunneling regime (*γ* = 1.33), Fig. 6(b) shows that Coulomb potential can increase the distribution for large longitudinal momentum for the rescattering electron nearly by a factor of two beyond *P*_*z*_ ~ 0.5 in this case. For weaker laser field and/or shorter wavelength, the quiver amplitude (*α* = *E*_0_/*ω*^2^) of the electron is much smaller, thus the electron is more prone to be pulled back to collide with the core in the presence of long-range potential. Figure 6(d) shows two sets of electron trajectories, each set with identical initial conditions (same ionization phase, tunneling position and initial momentum distribution) but different potential. It can be seen that Coulomb potential significantly modifies the motion as compared to a short range potential. This is understood that in the absence of a Coulomb potential, rescattering trajectories would be absent and thus electrons will emerge as direct electrons, causing significant drop in recollisions to contribute the formation of side lobes, as shown in Fig. 6(b).

## Discussion

In summary, a GQTMC model has been used to calculate the two-dimensional PMDs. The model was used to describe accurately the so-called side lobes which had been understood as photoelectron hologram. Like optical hologram, it is due to the interference between a reference wave and a signal wave. A hologram in principle offers the opportunity to probe the structure and dynamic information of the object. For side lobes considered here, the reference wave is the direct electron emission while the signal wave is due to electrons that have been rescattered by the ion. Unlike optical hologram, however, in photoelectron hologram, both the reference and signal waves are influenced by the subcycle ionization mechanism and by the subsequent interaction due to the laser field and the Coulomb potential. To use photoelectron holography to probe target structure, both of these effects have to be examined first. This is accomplished in the present work using the GQTMC method. In this method, the direct wave and signal wave can be separated and the structure can be extracted from the signal wave. Comparing the well-studied side lobes that are near the polarization axis in which the signal wave results from the emitted electrons colliding with the long range Coulomb potential of the ion, more useful target structure information is likely embedded in the side lobes that lie away from the polarization axis. These electrons come from hard collisions and emerge with large transverse momenta. The analysis from the present work can be extended to such large angle side lobes and may offer as a complementary tool for imaging the dynamics of structural changes at femtosecond timescale, similar to laser induced electron diffraction (LIED)^37^,^38^,^39^. The LIED method used backscatted photoelectrons in the high-energy region where there are no contributions from direct waves. The method is easier to analyze but it suffers from low yields. Photoelectron holography method would use lower energy electrons where ionization yield is much higher. Using GQTMC method, the direct wave can be extracted from the full spectrum. Further analysis of the signal wave would allow the determination of target structure. To make such a method possible, clearly photoelectron holography should first be investigated on molecular targets. It is also worth mentioning that photoelectron holography by long wavelength lasers discussed here is similarly used in ionization of molecules by hard X-rays. In this case, a hard X-ray is used to ionize the inner shell of a specific atom in the molecule. The photoelectrons generated may reach the detector directly, or via scattering with neighboring atoms. The interference of these two waves offers the opportunity to extract the target molecules. This method is being explored with X-ray free-electron lasers (XFEL’s)^40^ and a method for retrieving structure information had been proposed recently^41^.

## Methods

In the QTMC method, ionization is based on subcycle adiabatic tunneling ionization theory of Ammosov, Delone and Krainov (ADK)^33^, classical electron dynamics in the combined laser and Coulomb fields^21^,^22^,^23^, and Feynman’s path integral approach^24^,^42^. The ADK ionization rate is given by
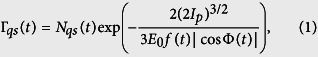
where
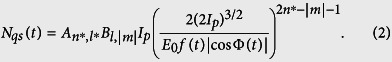


Here, *E*_0_ is the peak field, *f*(*t*) is the envelope function, and 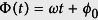 is the phase of the laser field. The *l* and *m* are the orbital angular momentum and its projection along the direction of polarization, respectively, 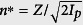 (*Z* is the ion charge, *I*_*p*_ is the ionization potential) and l^*^ are the effective principal quantum number and the effective orbital angular momentum of the initial state of the atom. The coefficient 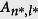 and 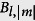 are
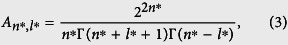
and
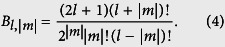


The ADK ionization rate is derived based on the adiabatic approximation, which means that the barrier does not move during tunneling. However, for *γ* ~ 1, the optical period, or the period of the oscillation of the barrier, is comparable to the time it takes for an electron to hit the barrier. In such cases, the quasi-static approximation is no longer valid. Thus non-adiabatic tunneling effect must be considered. In the GQTMC method, the static ionization rate is replaced by the Yudin-Ivanov formula:
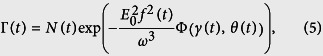
where
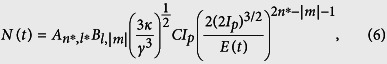
and
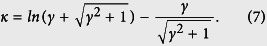


The coefficient, 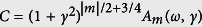, is the PPT correction to the quasistatic limit 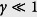. 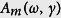 is given by:
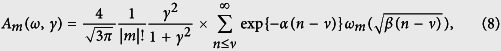

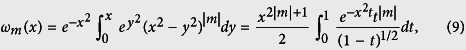
where 
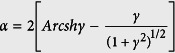
, 
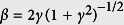
 and 
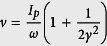
 are functions of the laser frequency and the electric field strength. The phase *θ*(*t*) is the defined as 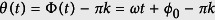 with the integer 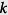 chosen to ensure that 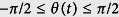.

These equations show that the main difference between ADK and Yudin-Ivanov ionization rates is in the exponential factors in Eqs (1) and (5). In the static ADK formula, the exponential factor depends on the instantaneous time, while in Yudin-Ivanov formula, the exponential factor is given by two functionals of time. The functionals give the distribution of ionization rate at each instant to account for nonadiabatic tunneling effect. In the tunneling limit, 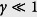, the Yudin-Ivanov rate is reduced to the ADK rate. As a result, GQTMC model has been applied to 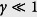, but also to *γ* ~ 1. By comparing GQTMC to QTMC results, we can clearly identify the role of nonadiabatic tunneling ionization to the side lobe spectra.

Following ionization, the evolution of the electron wave packet is simulated by launching randomly a set of electron trajectories with different initial conditions. The classical motion of an electron in the combined laser and Coulomb fields is governed by Newton equation:
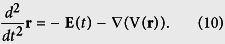


Here, V(**r**) is the potential of the ion. Each electron trajectory is weighted by the ionization rate 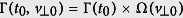 in which 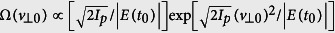 with the initial instantaneous field *E*(*t*_0_). Note that “⊥” is the direction perpendicular to the laser polarization axis.

In addition to modification of the ionization rate, we also use the more accurate tunnel exit point given by
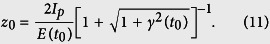


Here, *γ*(*t*_0_) is the Keldysh parameter depending on the instantaneous time. It is noticed that the exit point shifts toward the atomic core due to the nonadiabatic effect^34^. According to Feynman’s path integral approach, the phase of the jth electron trajectory in the ensemble is given by the classical action along the trajectory: 24,42
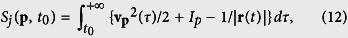
where *p* is the asymptotic momentum of the electron. The probability of each asymptotic momentum is determined by
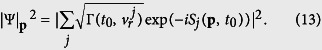


Using a parallel algorithm, the PMD was obtained with one billion electron trajectories.

## Additional Information

**How to cite this article**: Song, X. *et al*. Unraveling nonadiabatic ionization and Coulomb potential effect in strong-field photoelectron holography. *Sci. Rep.* **6**, 28392; doi: 10.1038/srep28392 (2016).
